# Development of an indirect ELISA to detect PEDV specific IgA antibody based on a PEDV epidemic strain

**DOI:** 10.1186/s12917-022-03419-w

**Published:** 2022-08-18

**Authors:** Kun Wang, Zhiqiang Hu, Mingyu Fan, Zhenwen Shao, Qiannan Yu, Xiaowen Li

**Affiliations:** 1 Shandong New Hope Liuhe Agriculture and Animal Husbandry Technology Co., Ltd (NHLH Academy of Swine Research), Dezhou, China; 2 Shandong Swine Health Data and Intelligent Monitoring Project Laboratory, Dezhou University, Dezhou, China; 3 Quality Control for Feed and Products of Livestock and Poultry Key Laboratory of Sichuan Province, New Hope Liuhe Co., Ltd, Chengdu, China

**Keywords:** PEDV, NH-TA2020, Indirect ELISA, IgA, IDEXX

## Abstract

**Background:**

Porcine epidemic diarrhea (PED), a swine epidemic disease caused by porcine epidemic diarrhea virus (PEDV), is characterized by severe watery diarrhea, vomiting, dehydration and high mortality in piglets, and has caused serious economic losses to the global porcine industry. The level of PEDV IgA antibody is a key marker to assess the extent of passive immunity of the resistance against PEDV infection. However, current commercial structure proteins-based kits for detection of PEDV antibody are not affordable, and those kits require complicated antigen preparation procedures, which cannot meet the scope of economic benefits of many large-scale pig companies in China. Therefore, there is an urgent need to develop an accurate, simple, and economical method for IgA detection in clinical samples. In this study, an indirect ELISA (i-ELISA) method was developed based on a purified PEDV epidemic strain (NH-TA2020).

**Results:**

The results show that optimal working dilution ratios of PEDV antigen and HRP anti-swine IgA are at 1: 1000 and 1:15000 respectively. The sensitivity of this method is high with the maximum dilution of samples up to 1:160, and coefficients of variation (CV) of both the intra assays and inter assays were no more than 15%. In addition, the relative sensitivities of the i-ELISA were above 90% compared with values from commercial kits in both serum and oral fluid samples.

**Conclusions:**

Our results suggested that the i-ELISA developed in this study was an accurate, simple, and economical method for PEDV-IgA detection in clinical samples.

**Supplementary Information:**

The online version contains supplementary material available at 10.1186/s12917-022-03419-w.

## Background

Porcine epidemic diarrhea (PED) is a highly contagious intestinal disease caused by porcine epidemic diarrhea virus (PEDV), which is characterized by severe watery diarrhea, vomiting and dehydration of piglets, causing huge economic losses to the global pig industry [1, 2]. PEDV, a member of the *Alphacoronavirus* genus within the *Coronaviridae* family, was first detected in Belgium in 1971 [3]. PEDV strains can be classified into two genotypes by phylogenetic analyses based on the whole genome: Genotype I and Genotype II [4]. The CV777 strain, a member of Genotype I first isolated in 1978, was epidemic in Europe, and reached South East Asia in the 1980s [3, 5]. Some highly virulent strains belong to Genotype II were reported and isolated in China in 2010, resulting in the more than one million death of piglets and the mortality rate of suckling piglets approaching 100% [6]. An inspection report of diarrhea samples in China collected from 2011 to 2014 showed that the positive rate of PEDV was about 61.10%-78.49%, higher than that of other diarrhea viral infection, indicating that PEDV was the major pathogen of swine viral diarrhea in China [7]. Low effectiveness of the prototype strain CV777-inactivated or related vaccines in many pig herds in China were observed, indicating variations of Chinese pandemic strains [6, 8]. It has been shown that variation of the virulence gene, S gene, can change the pathogenicity of PEDV [9, 10]. A molecular epidemiological investigation of Chinese PEDV strains from 2015 to 2018 identified 10 novel mutation positions of the S1 gene compared with PEDV CV777 strain and 10–11 novel mutation positions compared with 2011–2014 PEDV strains [11, 12]. These variations of S gene occurred gave more challenges to the prevention and diagnose of PEDV in China.

PEDV can infect pigs of all ages, but show lower pathogenicity in older pigs compared with neonatal pigs [13]. The protection of piglet is the most important factor to reduce the loss of PEDV outbreak. Currently, there is no specific drugs or effective vaccines against PEDV. The whole-herd feedback is an effective way before the applications of effective vaccines [14]. For example, Goede, D. et al. have reported one case that piglets born to a virulent PEDV isolate-exposed sow herd were all survived while piglets born to the control sow group had a 33% mortality rate [15]. Immunization of pregnant sows or gilts is undertaken by exposure to virulent autogenous virus, such as minced intestines from infected neonatal piglets negative for other infectious agents [14, 16]. Such feed-back loop stimulates neutralization antibody and PEDV-specific IgG or IgA antibody responses in serum 7–14 days later [13, 17, 18]. Although these systemic antibodies may contribute to PEDV clearance, protection of neonatal piglets can be attributed mostly to maternal secretory IgA (sIgA) antibodies [19, 20]. PEDV-specific sIgA antibody cannot be produced by mammary glands of female sows due to the absence of lymphoid follicles in mammary glands. However, it can be produced by effector B cells or plasmablasts that migrate from the gut-associated lymphoid tissue of the PEDV-infected intestine to the mammary gland and released to the colostrum and milk via the gut-mammary sIgA axis [19, 21]. Therefore, the levels of PEDV-specific sIgA antibody are keys to assess the extent of passive immunity of the PEDV resistance against PEDV infection in piglets [13].

ELISA is a convenient and rapid way for detection of the PEDV specific antibodies. In recent decades, three types of ELISA have been widely used to detect the level of PEDV antibodies in serum and colostrum, including indirect ELISA [22], competitive ELISA [23] and blocked ELISA [24]. The indirect ELISAs are developed based on recombinant viral structural proteins, including N protein [25], S protein [26, 27], and M protein [28]. The PEDV competitive or blocking ELISAs are based on the use of PEDV-specific monoclonal or polyclonal antibodies and have higher specificity compared to the indirect ELISA [29, 30]. The procedures of the antigen preparation in the methods mentioned here are relatively complicated and not cost effective, therefore, many loss-making companies or farms reduce application of these methods, especially in the period of African swine fever virus (ASFV) outbreak in China.

PEDV NH-TA2020 strain was an epidemic strain isolated from a PEDV-positive pig in Northern China in 2020. This strain showed a high progeny virus production in Vero cells in the laboratory. Here, taking advantage of the NH-TA2020 strain, we developed an i-ELISA method to detect IgA levels in clinical samples from pig farms. The viral antigen is easy to get and the i-ELISA method is suitable to evaluate the level of PEDV IgA antibody in different types of samples, including serum, oral fluid and colostrum. Our results showed similar sensitivity of i-ELISA compared to that in the commercial kit.

## Results

### PEDV antigen propagation and Purification

**Fig. 1:**
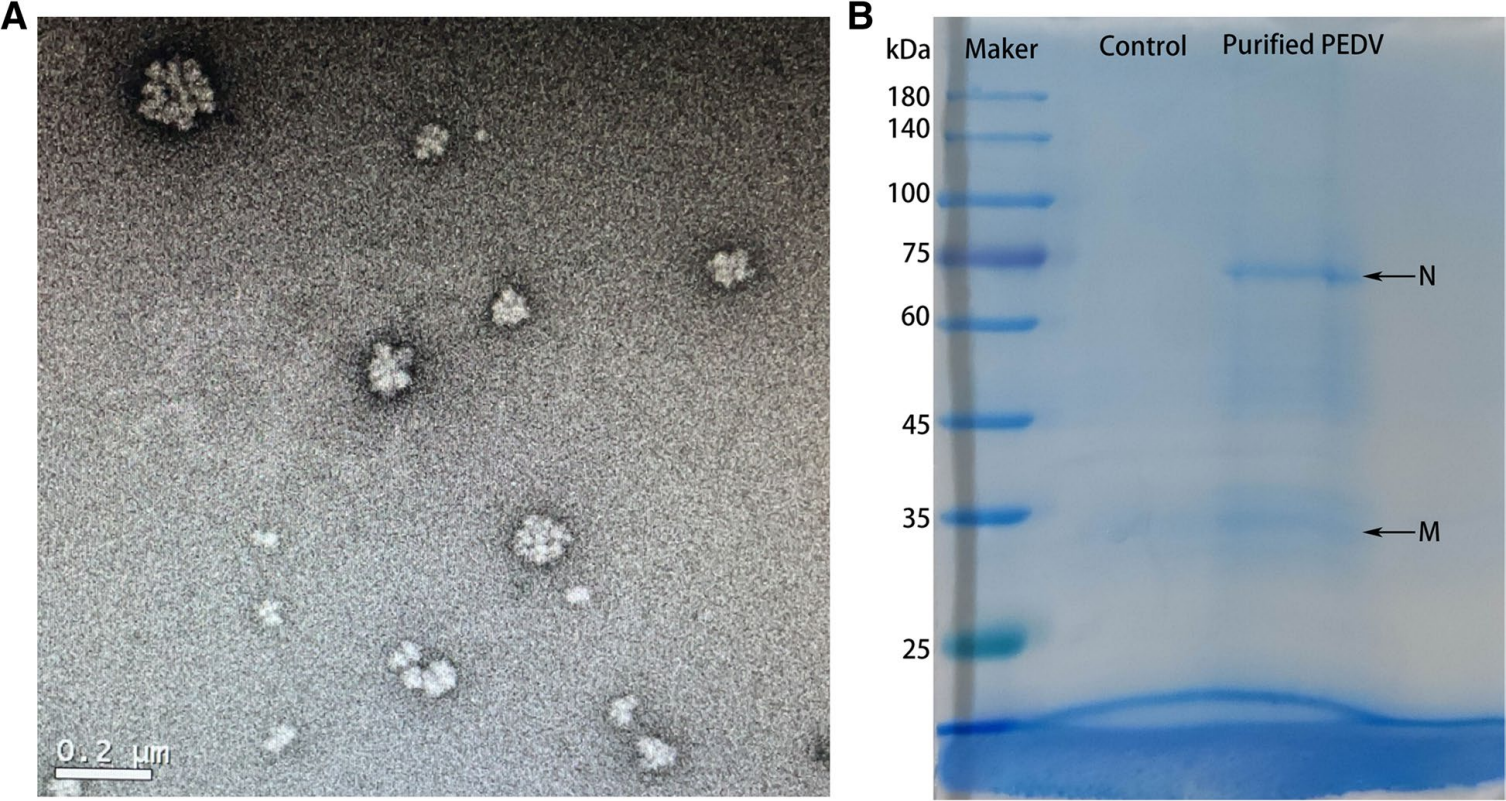
Purification of the whole-virus PEDV antigen. **A** The observation of purified PEDV particles by TEM, scale bar: 0.2 μm; **B** The detection of PEDV structural proteins by SDS-PAGE analysis, including N and M proteins (*Black arrows*). The ultracentrifuge products from PBS-adsorbed Vero cells were as the control

PED antigen was purified using the method of sucrose density-gradient ultracentrifugation. To verify the purification of PEDV antigens, we first used TEM to observe PEDV virions. As shown in Fig. 1A, many virus-like particles were observed, and the diameters of these particles were from 100 to 200 nm, which was consistent the literature [16, 31]. Furthermore, in the SDS-PAGE gel (Fig. 1B), there were two distinct bands, one at the position of near 75 kDa and the other one at the position near 35 kDa, which were consistent with the sizes of PEDV-N protein [32] and PEDV-M protein [33] respectively. Whereas, there were no bands observed in the ultracentrifuge products from PBS-adsorbed Vero cells. The raw image of Fig. 1B was shown in Supplementary Information file 1. Therefore, PEDV antigens were purified successfully and suitable to use in next steps.

### Optimization of working conditions

**Fig. 2:**
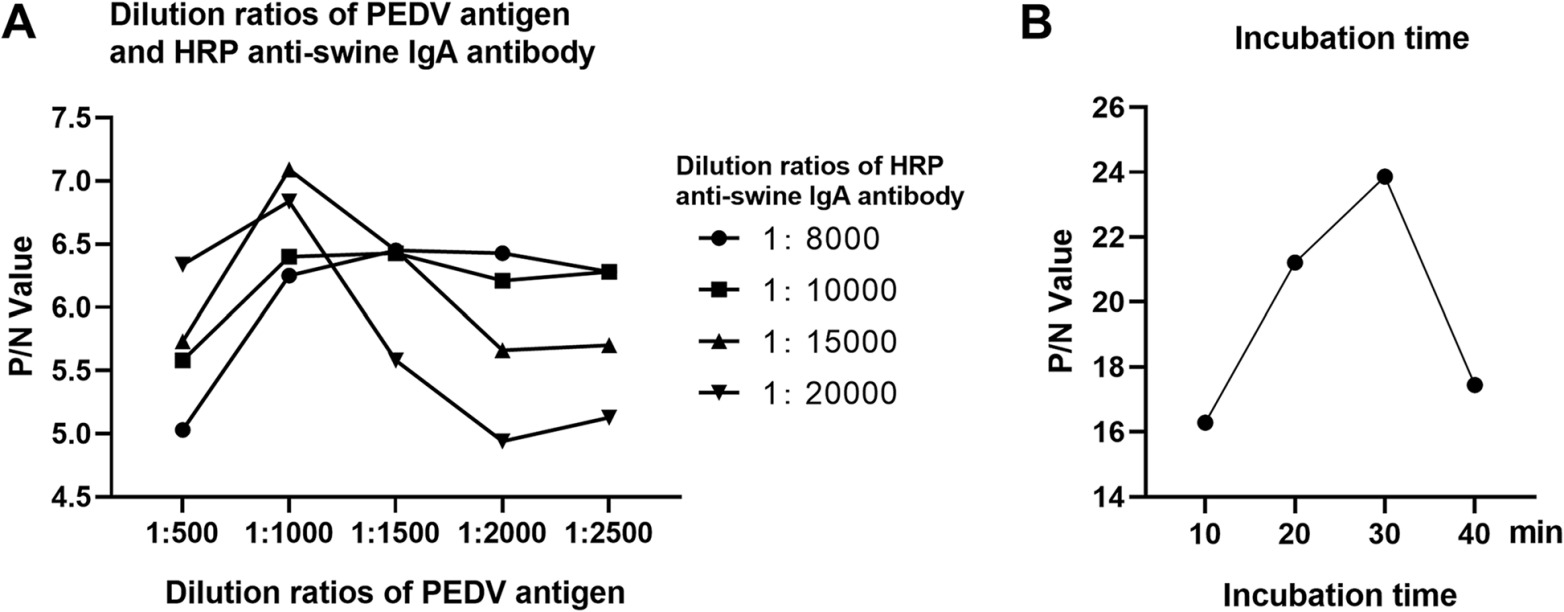
Optimization of working conditions. **A** Results of P/N values at different dilution ratios of PEDV antigen and HRP anti-swine IgA antibody. **B** Results of P/N values at different incubation times of samples with PEDV antigens

The OD450 ratios between the positive and negative samples (P/N values) with different dilution ratios of PEDV antigen and HRP anti-swine IgA antibody were shown in Fig. 2A. The maximum value of P/N was 7.09 with the dilution ratios of PEDV antigen at 1:1000 and HRP anti-swine IgA at 1:15000. The optimal incubation time were analyzed in Fig. 2B and the maximum value of P/N was 23.863 at the incubation time of 30 min. To sum up, the optimal working conditions of the i-ELISA method were with the dilution ratios of antigen and antibody at 1:1000 and 1:15000 respectively, and with the incubation time between samples and antigen for 30 min.

### Optimization of the dilution of samples

**Fig. 3:**
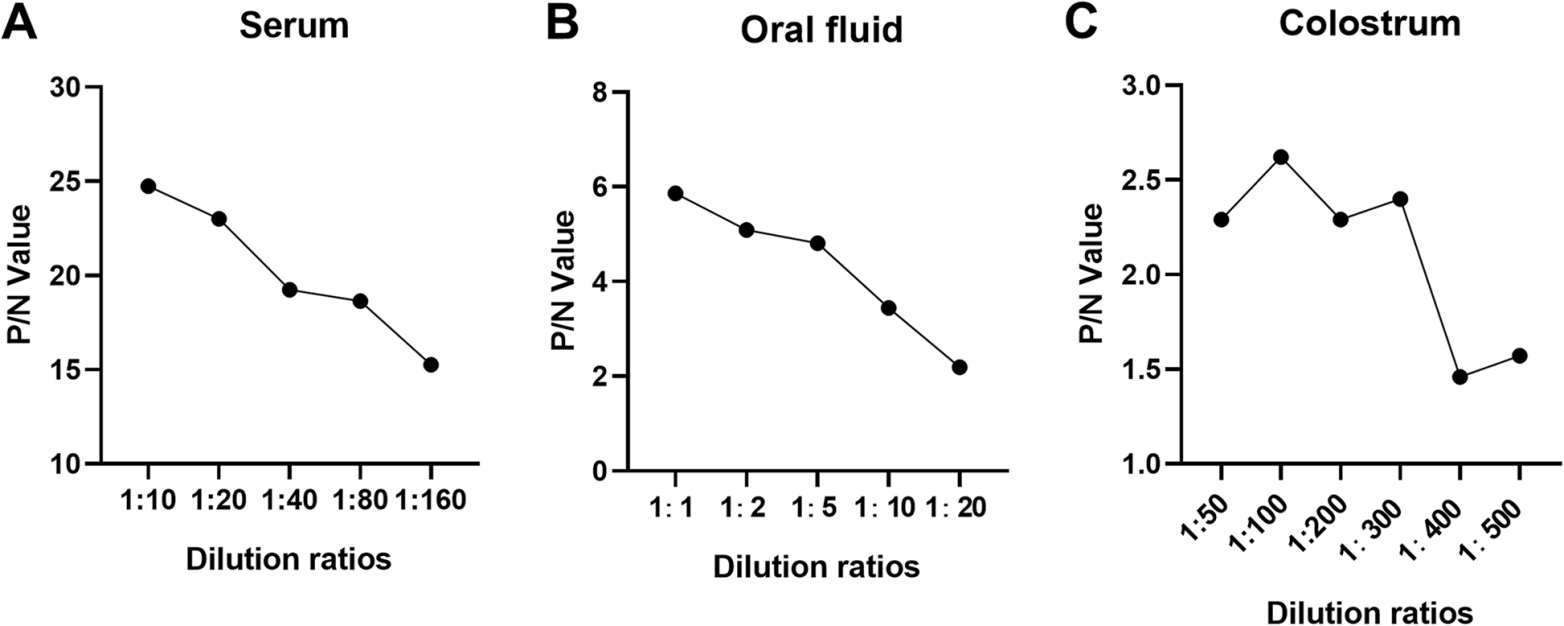
Optimization of dilution ratios of different types of samples. Results of P/N values of serum **(A)**, oral fluid **(B)** and colostrum **(C)** with different gradient dilutions respectively

As shown in Fig. 3, the maximum P/N values of serum, oral fluid and colostrum, were 24.74 at the dilution of 1:10 (Fig. 3A), 5.89 at the dilution of 1:1 (Fig. 3B), and 2.62 at the dilution of 1:100 (Fig. 3C) respectively. Therefore, the optimal working dilutions of serum, oral fluid and colostrum were 1:10, 1:1 and 1:100 respectively.

### The determination of cut-off value

As shown in Table 1, the results from PEDV antibody-negative samples, including serum (*N* = 160), oral fluid (*N* = 128) and colostrum (*N* = 68), showed the cut-off values of serum, oral fluid and colostrum were 0.361, 0.336 and 0.606 ($\overline{X }$ + 3SD). Therefore, serum samples with OD450 values ≥ 0.361 were considered as positive, while samples with OD450 values < 0.361 were considered negative. Oral fluid samples with OD450 values ≥ 0.336 were considered as positive, whereas samples with OD450 values < 0.336 were considered negative. Colostrum samples with OD450 values ≥ 0.606 were considered as positive while samples with OD450 values < 0.606 were considered negative.

**Table 1:** Determination of cut-off values of the i-ELISA in different sample types

Sample type	X	SD	Cut-off value
Serum	0.170	0.063	0.361
Oral fluid	0.102	0.078	0.336
Colostrum	0.303	0.101	0.606

### The evaluation of sensitivity and specificity

**Fig. 4:**
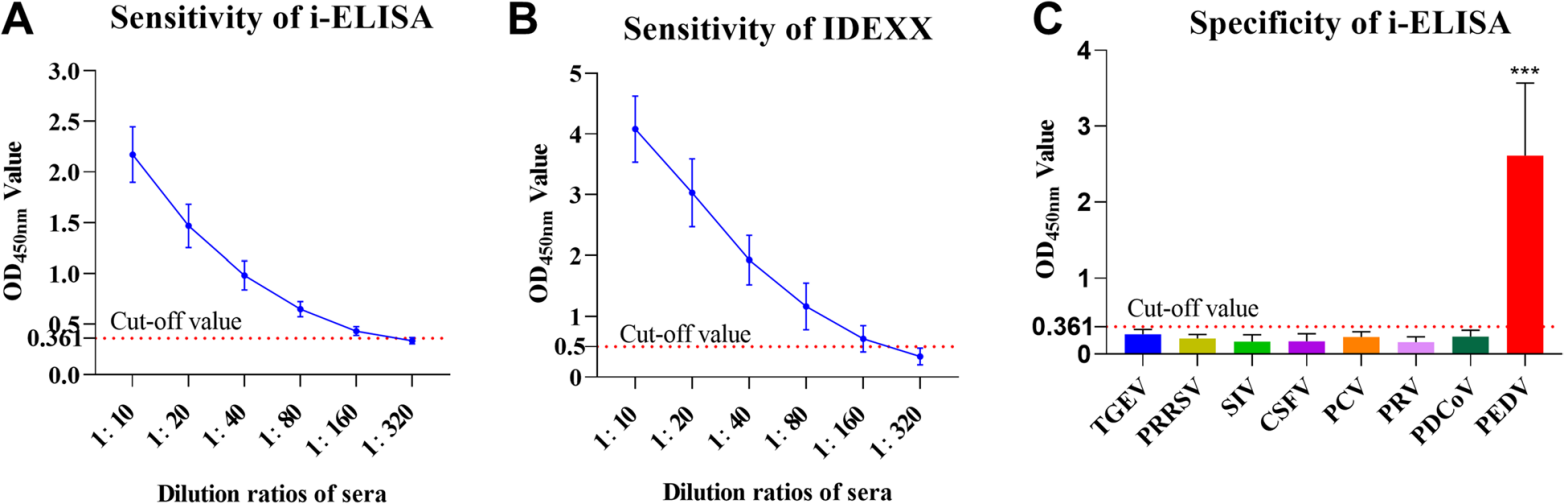
Sensitivity and specificity of the i-ELISA. (**A** and **B**) OD_450nm_ values of the positive serum with different dilution ratios were tested by both i-ELISA (**A**) and IDEXX (**B**) to determine the sensitivities; (**C**) Samples containing antibodies against other seven porcine viruses were test by the i-ELISA to determine the specificity. ***p < 0.001

PEDV-positive serum was diluted in double proportions and tested both by i-ELISA and a commercial kit (IDEXX). As shown in Fig. 4A and B, serum samples were diluted at the maximum dilution ratio of 1:160, which was similar to that of IDEXX kit. The results indicated that the sensitivity of i-ELISA is as good as IDEXX kit. The i-ELISA method was used to detect serum samples with antibody positive of porcine transmissible gastroenteritis virus (TGEV), Porcine reproductive and respiratory syndrome virus (PRRSV), Swine influenza virus (SIV), Classical Swine Fever Virus (CSFV), porcine circovirus (PCV) and Pseudorabies virus (PRV), porcine deltacoronavirus (PDCoV), but negative of PEDV. As shown in Fig. 4C, all pathogen-positive serum tested negative except for the 40 PEDV antibody-positive samples, indicating no cross-reactivity with other common swine pathogens.

### Evaluation of coefficient of variation (CV)

As shown in Table 2, the ranges of CV value were from 2.29% to 8.61% in intra-plate repetition and from 6.44 to 11.44% in inter-plate repetition, which were all no more than 15% and indicated that the established i-ELISA had good repeatability.

**Table 2:** Results of the repeatability assay for the i-ELISA

	**The range of CV value**	**Median value**
Intra-plate repetition (CV%)	2.29–8.61	7.49
Inter-plate Repetition (CV%)	6.44–11.44	9.45

### Comparison between i-ELISA and a commercial kit with clinical samples

**Fig. 5:**
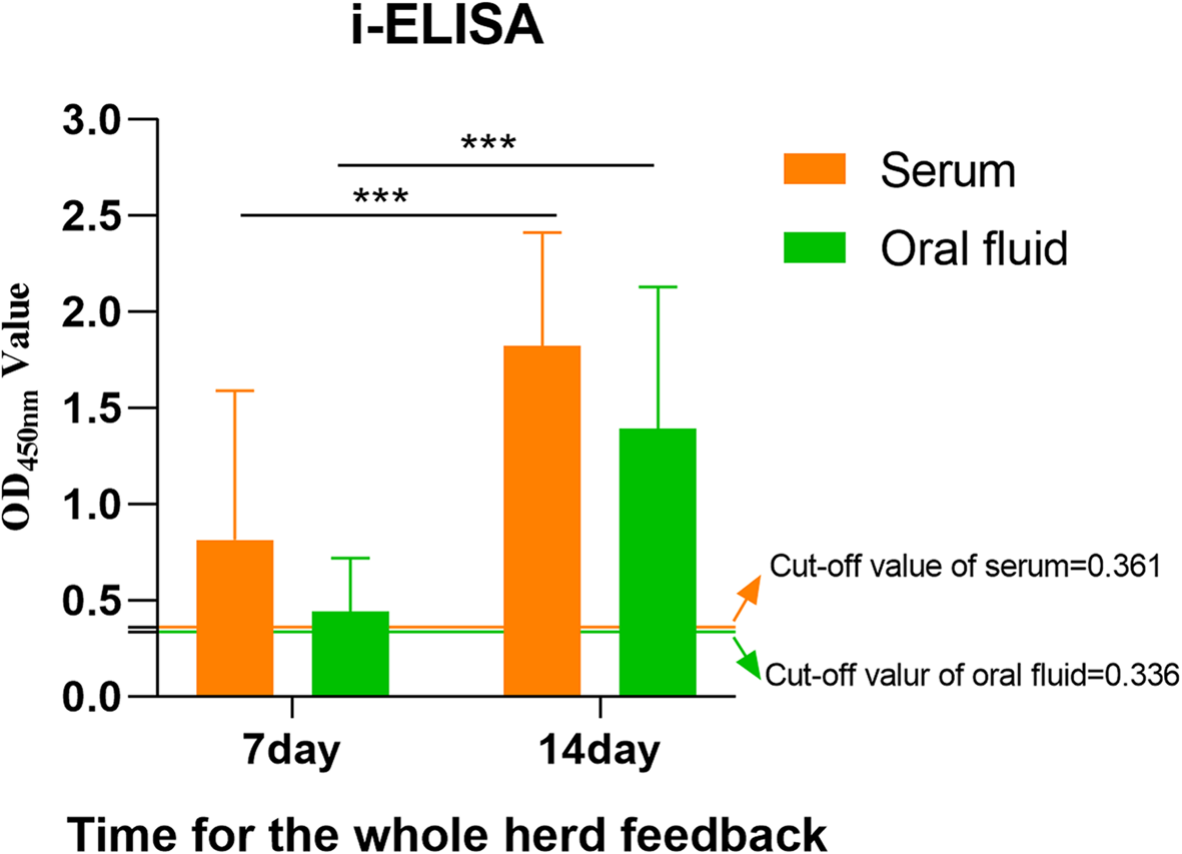
Determination of IgA levels of clinical samples by the i-ELISA. Serum (*N* = 40) and oral fluid (*N* = 40) samples were tested by the i-ELISA to evaluate changes of IgA levels in a sow herd with the whole-herd feedback treatment for 7 days and 14 days. ***p < 0.001

As shown in Tables 3 and 4, when samples were tested by both i-ELISA and IDEXX, the relative sensitivities of i-ELISA method were 93.33% and 91.67% in serum and oral fluid respectively, indicating high sensitivity compared with those of IDEXX kit. The relative specificities of i-ELISA method were 90.00% and 58.33%, indicating high specificity in serum samples while there is an improved specificity in oral fluid samples. The IgA levels of samples from a sow herd with the whole-herd feedback treatment were tested by the i-ELISA method (Fig. 5). There was a significant increase of IgA levels in serum samples from 7 to 14 days (*p* < 0.001), indicating a successful whole-herd feedback treatment. IgA levels of oral fluid samples increased significantly (*p* < 0.001), which was similar to findings found in serum samples.

**Table 3:** Comparison between i-ELISA and IDEXX with serum

		IDEXX		Total
		+	-	
i-ELISA	+	28	3	31
	-	2	27	29
Total		30	30	60
Relative sensitivity = 28/30 = 93.33%				
Relative specificity = 27/ 30 = 90.00%				
Compliance rate = 55/ 60 = 91.67%

**Table 4:** Comparison between i-ELISA and IDEXX with oral fluid

		IDEXX		Total
		+	-	
i-ELISA	+	33	10	43
	-	3	14	17
Total		36	24	60
Relative sensitivity = 33/36 = 91.67%				
Relative specificity = 14/ 24 = 58.33%				
Compliance rate = 47/ 60 = 78.33%

## Discussion

The current commonest method to monitor the level of PEDV antibody is ELISA. Most ELISA methods aim to detect IgG levels, and only a few mature methods were developed for detection of IgA [34]. The mucosal immunity in the oral cavity and intestine plays a major antiviral role in PEDV infection [35]. When pregnant sows are inoculated with PEDV vaccine, intestinal mucosal immunity is activated with a mass of IgA production. The secretory cells with IgA production migrate and colonize near the mammary gland of sows and release anti-PEDV specific IgA to colostrum/milk, which is received by newborn piglets to prevent PEDV from invading the body through intestinal epithelial cells [20, 36]. Therefore, IgA is important for prevention of PEDV for newborn piglets [37]. Detection of IgA level in the colostrum/milk and serum is necessary for sows and newborn piglets. Our company has developed a complete whole-herd feedback procedure, which can provide strong protection to piglets, but there is a lack of effective methods to evaluate the efficiency, especially for the regulation of IgA. Therefore, we further developed an indirect ELISA method to detect the level of anti-PEDV IgA based on the antigen of the whole virus.

The coated antigen (NH-TA2020) was a pandemic virulent strain circulating from a swine herd in Shangdong, China. The use of whole PEDV strain as the antigen in ELISA method has been proved to be an efficient way to test serum antibody IgG antibodies [38], which is more economical and practical than serum neutralization test [39]. To obtain purified virus particles, we used the method of sucrose density-gradient centrifugation, which was an improved method on the basis of Thomas JT’s method [38]. The consistency of morphological features of viral particles in Fig. 1A indicated the success of virus purification, and these particles seem like aggregations of several virions and were surrounded by black border areas, like the envelopes of coronavirus. The antigen preparation of this ELISA method was also simpler and more economical than that of PEDV structural protein-based methods [22, 25, 30, 39, 40].

This method could be used in different sample types, including serum, oral fluid and colostrum, and we have evaluated the optional working conditions of different sample types, which could support a clearer guideline for clinical application. The i-ELISA method could detect positive serum with PEDV antibody at a maximum dilution of 1:160. All six positive samples were tested positive by i-ELISA (6/6) at the dilution of 1:160, whereas only four samples tested positive by the IDEXX kit (4/6), indicating a better sensitivity of i-ELISA. Moreover, this method did not have cross-reaction with antibodies of some other pig virus, including TGEV, PRRSV, SIV, CSFV, PCV, PRV and PDCoV. The CV values both in intra-batch and inter-batch were no more than 15%, indicating high repeatability of this method. Furthermore, the relative sensitivity was both above 90% in serum and oral fluid compared to the IEDXX kit, indicating that this method can be used clinically. The relative specificities were 90.00% in serum while just 58.33% in oral fluid compared to the IEDXX kit, which might be due to complex components and impurities in oral fluid. Recently, much attention has been paid to PEDV-specific IgG and IgA antibodies detection in oral fluid samples as collection procedures of oral fluid were easier and faster, and even cheaper than that of collecting serum samples [41, 42]. Moreover, levels of IgA antibodies in oral fluid seem to increase for 100 days post-infection and may serve as a better marker to monitor the IgA levels of a whole sow herd [41, 42]. Another possible reason for relatively low specificity between i-ELISA and IDEXX in oral fluid samples was that the IDEXX kit was not suitable for IgA detection in oral fluid samples according to manufacturer’s instructions. Interestingly, our results showed that the trend of IgA changes in oral fluid was similar to that of serum in the monitorization of prior herd exposure to PEDV, indicating that the i-ELISA was suitable to detect IgA levels in oral fluid samples and monitor the effect of whole-feedback treatments.

In summary, the i-ELISA method developed this study was sensitive, specific, repeatable and suitable in testing antibody levels in serum, oral fluid and colostrum. It can serve as a simpler and cheaper method to monitor prior herd exposure to PEDV and assess the resistance of piglets to PEDV infection. Further application of this method in the detection of IgA levels in oral fluid samples provides a new idea for the prevention and control of PEDV in pig farms.

## Methods

### Viruses, cell lines, antibodies, chemicals and clinical samples

PEDV NH-TA2020 strain (CCTCC NO. V202097) was stored in our laboratory. Vero cells (Cat.No SCSP-520) were obtained from Cell bank of Chinese Academy of Sciences (Shanghai, China). HRP anti-swine IgA (H + L) (ab112746) were purchased from Abcam (Shanghai, China). TMB solution (PR1200) was purchased from Solarbio (Beijing, China). Serum, oral fluid and colostrum with or without PEDV infection were collected and stored in -80 ℃ in our laboratory.

### PEDV propagation and purification

Vero cells were incubated with Dulbecco’s modified Eagle’s medium (DMEM) with 0.05% (V/V) trypsin for 3 min and then replaced by PBS or PEDV stock for virus adsorption. After 1 h of absorption, cells were cultured with 3% (W/V) citric acid and DMEM with 1% Fetal bovine serum (FBS) (PAA, A15-151/101, Austria), and the final concentration of citric acid was 0.1% (W/V). Vero cells with progeny virus were harvested when cytopathic effect (CPE) reached over 80%. Each batch of one liter of PEDV propagated in harvested cells (infectious titers ranging from 10^6.0^–10^7.0^ TCID50 /mL) were subjected to freeze–thaw for three times and centrifuged at 8,000 × g for 30 min at 4 ℃ to remove cell debris. The supernatant was transferred to 32Ti centrifuge tube followed by ultracentrifugation at 100,000 × g for 2 h at 4 ℃. Then, the virus pellet was resuspended in 1 mL of sterile PBS and applied to a sucrose gradient of 20%, 40% and 60% (1.3 mL volume each) in 32Ti centrifuge tubes and centrifuged at 100,000 × g for 2 h at 4 ℃. The fractions on the border between 40 and 60% sucrose solutions were collected, resuspended in 4 mL PBS and ultracentrifuged at 100,000 × g for 2 h at 4 ℃ to remove sucrose. Finally, the pellet was resuspended in 400 μL sterile PBS and stored at -80 ℃ until use, and the protein concentration of the resuspended virus pellet in each batch was 2.0–2.4 mg/mL. Vero cells with PBS adsorption were as the control.

### SDS-PAGE

20 μL purified PEDV suspension were boiled for 5 min in 4 × SDS-PAGE loading buffer, and then were run by SDS-PAGE at 80 V for 30 min in stacking gels and 120 V for 90 min in 10% separating gels. The gel was subsequently stained with Coomassie Brilliant Blue Fast Staining solution (Solarbio, P1300, Beijing, China) according to the manufacturer’s instructions and imaged by ChemiDoc MP (Bio-Rad, Hercules, California, USA).

### Transmission electron microscopy (TEM)

The purified PEDV viruses were sent to Analytical & Testing Center, Sichuan University for TEM observation. Images were obtained with a JSM-7500F transmission electron microscope (JEOL).

### Optimization of working conditions

The dilution ratios of PEDV antigen and HRP anti-swine IgA antibody were optimized by checkerboard titration. The purified-PEDV antigen was diluted with carbonate buffer solution (CBS) by 1:500, 1:1000, 1:1500, and 1:2000, coated in an enzyme plate with 100 μL per well overnight at 4 ℃, sealed with 200 μL skim milk (5% W/V) at 37 ℃ for 1 h and washed with PBST for three times. Then, serum samples were added into the coated plate with 100 μL per well and incubated at 37 ℃ for 1 h. The HRP anti-swine IgA antibody was diluted by 1:8000, 1:10000, 1:15000, 1:20000, added into each well, incubated at 37 ℃ for 1 h, and washed with PBST for three times. Finally, the plate was incubated with TMB for 10 min and then read at OD value of 450 nm. The condition with the maximum P/N value was considered as the optimal antigen and antibody dilution ratios.

To find out the optimal incubation time of samples with PEDV antigens, the positive and negative serum samples were tested as the procedure above and the incubation time were 10 min, 20 min, 30 min and 40 min respectively. The condition with the maximum P/N value was considered as the optimal incubation time.

### Optimization of dilutions of samples

Serum samples with the dilution ratios of 1:20, 1:40, 1:60, 1:80, 1:160, oral fluid samples with the dilution ratios of 1:1, 1:2, 1:5, 1:10, and 1:20, and colostrum samples with the dilution ratios of 1:50, 1:100, 1:200, 1:300, 1:400 1:500 were tested by the i-ELISA. In addition, colostrum samples were left to stand for 4 h at 4 ℃ to remove fat and 100uL colostrum were then aspirated from the middle layer for diluting. All samples were diluted in skim milk (5% W/V) and the condition with the maximum P/N value was considered as the optimal working dilution.

### Determination of cut-off value

Negative serum samples (*N* = 160), negative oral fluid samples (*N* = 128) and negative colostrum (*N* = 68), were tested following i-ELISA procedure mentioned above, and the mean value ($\overline{X }$) and standard deviation (SD) of the OD450 values were calculated. The cut-off value was determined as $\overline{X }$ + 3SD.

### Evaluation of sensitivity and specificity

PEDV-positive serum was diluted by 1:20, 1:40, 1:80, 1:160 and 1:320, and tested by i-ELISA to determine the sensitivity. A commercial kit, IDEXX PEDV IgA (IDEXX, CAT# 99–55,550, Shanghai, China), was used as the control. PEDV antibody-negative while TGEV (*N* = 30), PRRSV (*N* = 26), SIV (*N* = 20), CSFV (*N* = 30), PCV (*N* = 22), PRV (*N* = 25) and PDCoV (*N* = 20) antibody-positive serum samples, as well as PEDV antibody-positive (*N* = 40) serum samples were tested by the i-ELISA to analyze the specificity.

### Evaluation of coefficient of variation (CV)

PEDV antibody-positive sera (*N* = 9) were randomly selected. Each sample with three replicates were assayed in the same plate to evaluate the intra-assay, and in three different plates to evaluate the inter-assay variation.

### Comparison between i-ELISA and a commercial kit with clinical samples

Clinical samples of serum (*N* = 60) and oral fluid (*N* = 60) from PEDV-positive farms were collected and tested by the i-ELISA method and a commercial kit. Results were compared with the commercial kit (IDEXX) to assess the performance of i-ELISA in terms of relative sensitivity [(true positive/(true positive + false negative)] ∗ 100% and relative specificity [(true negative/(true negative + false positive)] ∗ 100% [43].

In addition, serum (*N* = 40) and oral fluid (*N* = 40) samples were collected from a sow herd with the whole-herd feedback treatment [14] for 7 days and 14 days. The IgA levels of these samples were tested by the i-ELISA to assess the effect of the whole-herd feedback treatment.

### Statistical analysis

All data were analyzed by the two-tailed independent Student’s t-test using the GraphPad Prism software (version 8.0). A *P* value of < 0.05 was considered to be statistically significant.

## Supplementary Information

**Additional file 1.**

## Data Availability

The datasets generated and/or analyzed during the current study are not publicly available due to the reason that clinical samples used in this study were from commercial farms, and the data were only used under license for the current study, but are available from the corresponding author on reasonable request.

## Abbreviations

PED: Porcine epidemic diarrhea
PEDV: Porcine epidemic diarrhea virus
i-ELISA: Indirect ELISA
CV: Coefficients of variation
ASFV: African swine fever virus
DMEM: Dulbecco’s modified Eagle’s medium
CBS: Carbonate buffer solution

## References

[1] Trujillo-Ortega ME, Beltrán-Figueroa R, García-Hernández ME, Juárez-Ramírez M, Sotomayor-González A, Hernández-Villegas EN, Becerra-Hernández JF, Sarmiento-Silva RE (2016). Isolation and characterization of porcine epidemic diarrhea virus associated with the 2014 disease outbreak in Mexico: case report. BMC Vet Res.

[2] Gerber PF, Lelli D, Zhang J, Strandbygaard B, Moreno A, Lavazza A, Perulli S, Bøtner A, Comtet L, Roche M (2016). Diagnostic evaluation of assays for detection of antibodies against porcine epidemic diarrhea virus (PEDV) in pigs exposed to different PEDV strains. Prev Vet Med.

[3] Pensaert MB, de Bouck P (1978). A new coronavirus-like particle associated with diarrhea in swine. Adv Virol.

[4] Zhang Y, Chen Y, Yuan W, Peng Q, Zhang F, Ye Y, Huang D, Ding Z, Lin L, He H (2020). Evaluation of Cross-Protection between G1a- and G2a-Genotype Porcine Epidemic Diarrhea Viruses in Suckling Piglets. Animals.

[5] Williamson S, Strugnell B, Thomson J, Webster G, McOrist S, Clarke H (2013). Emergence of severe porcine epidemic diarrhoea in pigs in the USA. Veterinary Record.

[6] Li W, Li H, Liu Y, Pan Y, Deng F, Song Y, Tang X, He Q (2012). New Variants of Porcine Epidemic Diarrhea Virus, China, 2011. Emerg Infect Dis.

[7] Sun D, Wang X, Wei S, Chen J, Feng L (2016). Epidemiology and vaccine of porcine epidemic diarrhea virus in China: a mini-review. J Vet Med Sci.

[8] Wang J, Zhao P, Guo L, Liu Y, Du Y, Ren S, Li J, Zhang Y, Fan Y, Huang B (2013). Porcine epidemic diarrhea virus variants with high pathogenicity. China Emerg Infect Dis.

[9] Deng F, Ye G, Liu Q, Navid MT, Zhong X, Li Y, Wan C, Xiao S, He Q, Fu ZF (2016). Identification and Comparison of Receptor Binding Characteristics of the Spike Protein of Two Porcine Epidemic Diarrhea Virus Strains. Viruses.

[10] Lin C-M, Saif LJ, Marthaler D, Wang Q (2016). Evolution, antigenicity and pathogenicity of global porcine epidemic diarrhea virus strains. Virus Res.

[11] Wen Z, Li J, Zhang Y, Zhou Q, Gong L, Xue C, Cao Y (2018). Genetic epidemiology of porcine epidemic diarrhoea virus circulating in China in 2012–2017 based on spike gene. Transbound Emerg Dis.

[12] Su M, Li C, Qi S, Yang D, Jiang N, Yin B, Guo D, Kong F, Yuan D, Feng L (2020). A molecular epidemiological investigation of PEDV in China: Characterization of co-infection and genetic diversity of S1-based genes. Transbound Emerg Dis.

[13] Jung K, Saif LJ, Wang Q (2020). Porcine epidemic diarrhea virus (PEDV): An update on etiology, transmission, pathogenesis, and prevention and control. Virus Res.

[14] Niederwerder MC, Hesse RA (2018). Swine enteric coronavirus disease: A review of 4 years with porcine epidemic diarrhoea virus and porcine deltacoronavirus in the United States and Canada. Transbound Emerg Dis.

[15] Goede D, Murtaugh MP, Nerem J, Yeske P, Rossow K, Morrison R (2015). Previous infection of sows with a “mild” strain of porcine epidemic diarrhea virus confers protection against infection with a “severe” strain. Vet Microbiol.

[16] Jung K, Saif LJ (2015). Porcine epidemic diarrhea virus infection: Etiology, epidemiology, pathogenesis and immunoprophylaxis. Vet J.

[17] Annamalai T, Lin CM, Gao X, Liu X, Lu Z, Saif LJ, Wang Q (2017). Cross protective immune responses in nursing piglets infected with a US spike-insertion deletion porcine epidemic diarrhea virus strain and challenged with an original US PEDV strain. Vet Res.

[18] Chen Q, Thomas JT, Giménez-Lirola LG, Hardham JM, Gao Q, Gerber PF, Opriessnig T, Zheng Y, Li G, Gauger PC (2016). Evaluation of serological cross-reactivity and cross-neutralization between the United States porcine epidemic diarrhea virus prototype and S-INDEL-variant strains. BMC Vet Res.

[19] Langel SN, Paim FC, Lager KM, Vlasova AN, Saif LJ (2016). Lactogenic immunity and vaccines for porcine epidemic diarrhea virus (PEDV): Historical and current concepts. Virus Res.

[20] Langel SN, Paim FC, Alhamo MA, Buckley A, Van Geelen A, Lager KM, Vlasova AN, Saif LJ (2019). Stage of Gestation at Porcine Epidemic Diarrhea Virus Infection of Pregnant Swine Impacts Maternal Immunity and Lactogenic Immune Protection of Neonatal Suckling Piglets. Front Immunol.

[21] Langel SN, Wang Q, Vlasova AN, Saif LJ (2020). Host Factors Affecting Generation of Immunity Against Porcine Epidemic Diarrhea Virus in Pregnant and Lactating Swine and Passive Protection of Neonates. Pathogens.

[22] Gerber PF, Gong Q, Huang Y-W, Wang C, Holtkamp D, Opriessnig T (2014). Detection of antibodies against porcine epidemic diarrhea virus in serum and colostrum by indirect ELISA. Vet J.

[23] Sozzi E, Moreno A, Lelli D, Perulli S, Prosperi A, Brocchi E, Capucci L, Papetti A, Giacomini E, Alborali GL (2018). Development and validation of a monoclonal antibody-based competitive ELISA for detection of antibodies against porcine epidemic diarrhoea virus (PEDV). Res Vet Sci.

[24] Carvajal A, Lanza I, Diego R, Rubio P, Cámenes P (1995). Evaluation of a Blocking ELISA Using Monoclonal Antibodies for the Detection of Porcine Epidemic Diarrhea Virus and Its Antibodies. J Vet Diagn Invest.

[25] Hou XL, Yu LY, Liu J (2007). Development and evaluation of enzyme-linked immunosorbent assay based on recombinant nucleocapsid protein for detection of porcine epidemic diarrhea (PEDV) antibodies. Vet Microbiol.

[26] Li Y, Zheng F, Fan B, Muhammad HM, Zou Y, Jiang P (2015). Development of an indirect ELISA based on a truncated S protein of the porcine epidemic diarrhea virus. Can J Microbiol.

[27] Shan Y, Gao Q, Mao J, Zheng J, Xu X, Zhang C, Huang X, Xu J, Shi F, Yue M (2022). Establishment of enzyme-linked immunosorbent assays based on recombinant S1 and its truncated proteins for detection of PEDV IgA antibody. BMC Vet Res.

[28] Ren X, Suo S, Jang YS (2011). Development of a porcine epidemic diarrhea virus M protein-based ELISA for virus detection. Biotech Lett.

[29] Aydin S (2015). A short history, principles, and types of ELISA, and our laboratory experience with peptide/protein analyses using ELISA. Peptides.

[30] Okda F, Liu X, Singrey A, Clement T, Nelson J, Christopher-Hennings J, Nelson EA, Lawson S (2015). Development of an indirect ELISA, blocking ELISA, fluorescent microsphere immunoassay and fluorescent focus neutralization assay for serologic evaluation of exposure to North American strains of Porcine Epidemic Diarrhea Virus. BMC Vet Res.

[31] Oka T, Saif LJ, Marthaler D, Esseili MA, Meulia T, Lin CM, Vlasova AN, Jung K, Zhang Y, Wang Q (2014). Cell culture isolation and sequence analysis of genetically diverse US porcine epidemic diarrhea virus strains including a novel strain with a large deletion in the spike gene. Vet Microbiol.

[32] Wang X, Fang L, Zhan J, Shi X, Liu Q, Lu Q, Bai J, Li Y, Jiang P (2020). Identification and characterization of linear B cell epitopes on the nucleocapsid protein of porcine epidemic diarrhea virus using monoclonal antibodies. Virus Res.

[33] Utiger A, Tobler K, Bridgen A, Ackermann M (1995). Identification of the membrane protein of porcine epidemic diarrhea virus. Virus Genes.

[34] Gerber PF, Opriessnig T (2015). Detection of immunoglobulin (Ig) A antibodies against porcine epidemic diarrhea virus (PEDV) in fecal and serum samples. MethodsX.

[35] Krishna VD, Kim Y, Yang M, Vannucci F (2020). Cheeran CJJPO: Immune responses to porcine epidemic diarrhea virus (PEDV) in swine and protection against subsequent infection. PLoS One.

[36] Boonsoongnern P, Boodde O, Chumsing W, Sukmak M, Boonsoongnern AJVW (2021). Correlation between antibody response against porcine epidemic diarrhea virus in sows and their offspring under field conditions. Vet World.

[37] Suda Y, Miyazaki A, Miyazawa K, Shibahara T, Ohashi S (2021). Systemic and intestinal porcine epidemic diarrhea virus-specific antibody response and distribution of antibody-secreting cells in experimentally infected conventional pigs. Vet Res.

[38] Thomas JT, Chen Q, Gauger PC, Giménez-Lirola LG, Sinha A, Harmon KM, Madson DM, Burrough ER, Magstadt DR, Salzbrenner HMJPO (2015). Effect of Porcine Epidemic Diarrhea Virus Infectious Doses on Infection Outcomes in Nave Conventional Neonatal and Weaned Pigs. PLoS One.

[39] Oh JS, Song DS, Yang JS, Song JY, Moon HJ, Kim TY, Park BK (2005). Comparison of an enzyme-linked immunosorbent assay with serum neutralization test for serodiagnosis of porcine epidemic diarrhea virus infection. J Vet Sci.

[40] Paudel S, Park JE, Jang H, Shin HJ (2014). Comparison of serum neutralization and enzyme-linked immunosorbent assay on sera from porcine epidemic diarrhea virus vaccinated pigs. Veterinary Quarterly.

[41] Bjustrom-Kraft J, Woodard K, Giménez-Lirola L, Rotolo M, Wang C, Sun Y, Lasley P, Zhang J, Baum D, Gauger P (2016). Porcine epidemic diarrhea virus (PEDV) detection and antibody response in commercial growing pigs. BMC Vet Res.

[42] Diel DG, Lawson S, Okda F, Singrey A, Clement T, Fernandes MHV, Christopher-Hennings J, Nelson EA (2016). Porcine epidemic diarrhea virus: An overview of current virological and serological diagnostic methods. Virus Res.

[43] Zhong K, Zhu M, Yuan Q, Deng Z, Feng S, Liu D, Yuan X (2022). Development of an Indirect ELISA to Detect African Swine Fever Virus pp62 Protein-Specific Antibodies. Front Vet Sci.

