# Supplementary figures and images for: Development of an indirect ELISA to detect PEDV specific IgA antibody based on a PEDV epidemic strain
Source: BMC Vet Res. 2022 Aug 18;18:319. doi: 10.1186/s12917-022-03419-w (PMC9386190; doi:10.1186/s12917-022-03419-w)

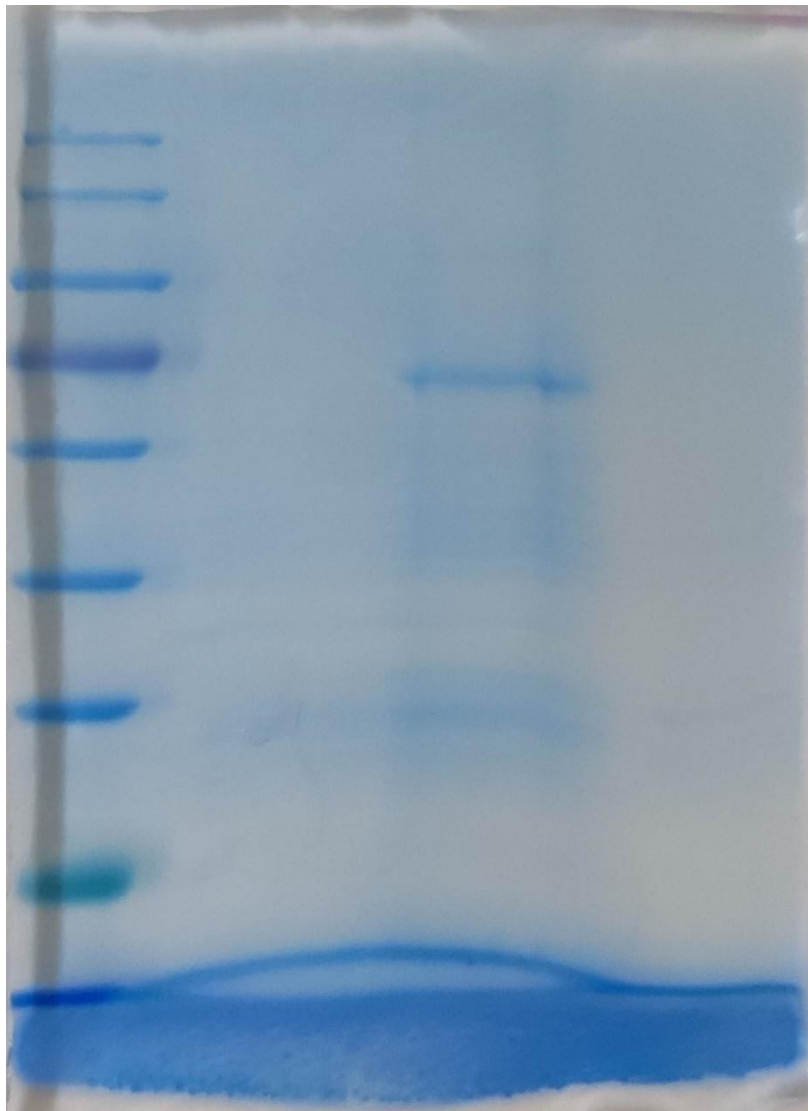

The raw image of Figure 2B

Supplement: Supplementary file 1 — Additional file 1. [file 12917_2022_3419_MOESM1_ESM.pdf]
